# Network pharmacology reveals the potential mechanism of Baiying Qinghou decoction in treating laryngeal squamous cell carcinoma

**DOI:** 10.18632/aging.203786

**Published:** 2021-12-20

**Authors:** Kun Gao, Yanan Zhu, Hui Wang, Xianwei Gong, Zhiyong Yue, Aiai Lv, Xuanchen Zhou

**Affiliations:** 1 Department of Otorhinolaryngology Head and Neck Surgery, Shandong Provincial Hospital Affiliated to Shandong First Medical University, Jinan 250021, Shandong, China; 2 Department of Otorhinolaryngology Head and Neck Surgery, Shandong Provincial Hospital Affiliated to Shandong University, Jinan 250021, Shandong, China; 3 Department of Internal Medicine, Shandong Provincial Chest Hospital Affiliated to Shandong University, Jinan 250013, Shandong, China; 4 Department of Ultrasound, The Fifth People’s Hospital of Jinan, Jinan 250022, Shandong, China; 5 Department of Pharmacy, Shandong Provincial Hospital Affiliated to Shandong First Medical University, Jinan 250021, Shandong, China

**Keywords:** Baiying Qinghou decoction, laryngeal squamous cell carcinoma, molecular docking, network pharmacology, protein-protein interaction

## Abstract

Context: Baiying Qinghou as a traditional Chinese medicine decoction shows anticancer property on laryngeal squamous cell carcinoma. However, little is known about the precise mechanism of Baiying Qinghou detection against laryngeal squamous cell carcinoma.

Objective: This study was aimed to explore potential mechanism of therapeutic actions of Baiying Qinghou decoction on laryngeal squamous cell carcinoma.

Materials and Methods: The active chemical components of Baiying Qinghou decoction were predicted, followed by integrated analysis of network pharmacology and molecular docking approach. The network pharmacology approach included target protein prediction, protein-protein interaction network construction and functional enrichment analysis.

Results: Sitosterol and quercetin were predicted to be the overlapped active ingredients among three Chinese herbs of Baiying Qinghou decoction. The target proteins were closely associated with response to chemical, response to drug related biological process and cancer related pathways such as PI3K-Akt signaling, HIF-1 signaling and Estrogen signaling pathway. The target proteins of *TP53*, *EGFR*, *PTGS2*, *NOS3* and *IL1B* as the key nodes in PPI network were cross-validated, among which *EGFR*, *IL1B*, *NOS3* and *TP53* were significantly correlated with the prognosis of patients with laryngeal squamous cell carcinoma. Finally, the binding modes of *EGFR*, *IL1B*, *NOS3* and *TP53* with quercetin were visualized.

Discussion and Conclusion: Quercetin of Baiying Qinghou decoction showed therapeutic effect against laryngeal squamous cell carcinoma by regulating *TP53*, *EGFR*, *NOS3* and *IL1B* involved with drug resistance and PI3K-AKT signaling pathway. *TP53*, *EGFR*, *NOS3* and *IL1B* may be the candidate targets for the treatment of laryngeal squamous cell carcinoma.

## INTRODUCTION

Head and neck cancer (HNC) is the seventh most common malignant tumor worldwide and occurs in nasopharynx, larynx, and thyroid [1, 2]. Head and neck squamous cell carcinoma (HNSCC) accounts for over 90% cases of head and neck cancer (HNC) and mainly derives from the mucosal surfaces of the upper aerodigestive tract [3]. Laryngeal squamous cell carcinoma (LSCC) represents the second most common histological subtypes of HNC with an increasing incidence rate [4]. Patients with LSCC are usually diagnosed at a late clinical stage and the survival rate is lower due to regional or distant metastases. Thus, LSCC has caused noticeable medical and economic burden worldwide.

Currently, several mainstream options against LSCC include surgery, radiotherapy, chemotherapy and chemo-radiotherapy [5]. However, the overall survival of patients with LSCC has not been remarkably improved because of chemotherapy or drug resistance and undesired effects. Traditional Chinese medicines (TCM) has been considered as an attractive alternative therapy for conquering cancers in China [6]. TCM focuses on restoring body balance and boosting immunity by the synergistic effects of various active ingredients [7]. Some Chinese herbs have been frequently utilized for the treatment of different diseases, such as cardiovascular diseases, diabetes and caners [8–10]. A recent research highlights that erchen plus huiyanzhuyu decoction can inhibit the growth of laryngeal carcinoma by modulating STAT3/cyclin D1 signaling pathway [11]. TCM exerts promising therapeutic effect on various malignant tumors.

Network pharmacology is proposed by Hopkins et al. and aims to explore the multilevel interactions of diseases, genes, and drugs as a whole [12]. This systems pharmacology is based on systems biology, computational biology and omics theory to evaluate the therapeutic effects of Chinese medicines on several diseases [13]. Gao et al. indicate that 8 herbs regulate multiple hepatocellular carcinoma-related genes and are strongly associated with prognosis by a network pharmacology approach [14]. Huang et al. suggest Huanglian Jiedu decoction has therapeutic roles in cancers such as hepatocellular carcinoma by building the herb-compound, compound-protein, protein-pathway, and gene-disease networks [15]. Notably, Bai Ying Qing Hou (BYQH) decoction is a Chinese medicinal formula and widely used for the treatment of LSCC in China, which is primarily composed of five key traditional Chinese herbs: Solanum lyratum thumb (30 g), Scutellaria barbata (24 g), Duchesnea indica (24 g), Solanum nigrum (30 g), and Actinidia chinensis planch (30 g). However, the pharmacological mechanism of BYQH decoction in the treatment of LSCC has not been clarified.

In this study, we attempted to explore the mechanism of BYQH decoction in the treatment of LSCC by network pharmacology analysis. First, the key active chemical constituents and their target proteins of BYQH decoction were screened. Then, the Gene Ontology (GO) and Kyoto Encyclopedia of Genes and Genomes (KEGG) pathway analyses of gene targets were carried out. Besides, protein-protein interaction (PPI) and target proteins were cross-validated followed by survival analysis. Finally, molecular docking was used to identify the binding mode of key active ingredients and target proteins of BYQH decoction.

## MATERIALS AND METHODS

### Screening active constituents of BYQH decoction

The traditional Chinese medicine systems pharmacology database (TCMSP) is an herbal repository and provides the system information about Chinese herbal medicines [16]. The absorption, distribution, metabolism, and excretion (ADME) system has been successfully used to evaluate the pharmacokinetics characteristics of chemical compounds [17, 18]. Herein, the chemical composition of five key components of BYQH decoction was firstly searched from TCMSP, mainly including the number of the pharmaceutical ingredient, molecule name, molecular weight, lipid-water partition coefficient, the number of hydrogen-bonding donor or acceptor, oral bioavailability (OB), blood brain barrier, Caco-2 permeability (Caco-2), drug-likeness (DL), and half-life (HL). OB ≥ 30% and Caco-2 ≥ −0.4 indicate good OB and permeability of molecules. The mean DL value of drugs collected in database is 0.18. Then, active chemical compounds were screened in ADME system according to the criteria of OB ≥ O30%, DL ≥ 0.18 and Caco-2 ≥ −0.4 according the previous description [19].

### Screening protein targets of active chemical compounds

TCMSP and DrugBank (https://www.drugbank.ca/) databases can predict the relationships between drugs and corresponding targets. Moreover, DrugBank database provides the detailed information on experimental and investigational drugs [20]. The active chemical compounds were subjected to TCMSP and DrugBank databases to search their potential targets. Then, the target proteins were mapped to corresponding gene symbols using string database. For those proteins that did not map to any gene symbols, they were manually retrieved by Universal Protein (UniProt) database to map to corresponding gene symbols. These potential gene targets were used for following analysis.

### Functional enrichment analysis of target genes

To investigate the biological function of the target genes, the gene ontology (GO) functional annotation and Kyoto Encyclopedia of Genes and Genomes (KEGG) pathway enrichment analyses were carried out. There are three categories for GO terms, including biological process (BP), cellular component (CC) and molecular function (MF). Gene symbols were input into string database. The parameters were set as the default values. The significantly enriched GO and pathway terms with *p* < 0.05 were collected. Then, the top 10 GO terms in each category and top 10 significant pathways were visualized in bubble chart by using R language.

### Target protein-protein interaction (PPI) analysis

The underlying relationships among target proteins were analyzed based on the string database according to the default parameters [21]. The Cytoscape software was used to construct and visualize the PPI network. Following this, the topological characteristics of the PPI network were also evaluated. The node degree (connectivity) was calculated.

### Cross-validation of target proteins by the database retrieval

DisGeNET is a versatile platform that comprehensively collects human gene-disease associations (over 380,000 associations between more than 16,000 genes and 13,000 diseases) for the validation of computationally predicted genes for human diseases [22, 23]. In this study, the genes related to LSCC were firstly searched from this database using “carcinoma of larynx” as the keyword. Phenopedia database also provides systematical genetic association studies and summarizes the information about the association between genes studied and a particular disease. Therefore, the known genes associated with LSCC were obtained by retrieving Phenopedia database with the keyword of “laryngeal neoplasms”. Subsequently, the overlapping genes among target genes of BYQH decoction, LSCC-related genes in DisGeNET or Phenopedia database were extracted by jvenn tool [24, 25]. These genes may be associated with the therapeutic effects of BYQH decoction against LSCC.

### Survival analysis of candidate genes

Firstly, expression profiles of HNSCC samples deposited in the Cancer Genome Atlas (TCGA) dataset were downloaded from UCSC Xena (https://xenabrowser.net/datapages/). Based on the clinical information, the HNSCC samples were selected according to following criteria: 1) samples were primary tumor tissues and the primary site was at the larynx; 2) the clinical information of samples with regard to TNM stage, clinical stage, grade and follow-up were complete; 3) the samples without survival time were deleted. Finally, a total of 105 laryngeal cancer samples were retained for the next prognostic analysis.

To investigate whether key candidate genes are associated with prognosis of patients with laryngeal cancer, the survival analyses of these gene targets were carried out. In brief, the raw data of 105 laryngeal cancer samples were firstly extracted and then standardized by log_2_ (count+1). After that, the optimal cutoff was determined using survminer package in R language based on the expressions of candidate genes. Subsequently, the samples were divided into high- and low- expression group according to the optimal cutoff. Following this, the survival analyses of gene targets were respectively preformed with survival package in R language. *P* < 0.05 was considered significant.

### Predicting the binding mode between active compounds and key protein targets of BYQH decoction

The Protein Data Bank (PDB) offers the experimental data for determined three-dimensional (3D) biological macromolecules structure [26]. Herein, we would further explore the underlying molecular mechanism how active compounds interacted with target proteins of BYQH decoction. The PDB ID for the key protein was firstly acquired from PDB. Subsequently, the binding mode between key protein and corresponding active chemical ingredient was predicted by the LeDock tool. Finally, we used the Pymol software to visualize the predicted binding modes of key active ingredients to their corresponding targets.

### Ethics approval and consent to participate

This study was approved by Ethics Committee of Provincial Hospital Affiliated to Shandong First Medical University and Shandong Provincial Hospital affiliated to Shandong University.

### Availability of data and materials

The raw data supporting the conclusions of this manuscript will be made available by the authors, without undue reservation, to any qualified researcher.

### Highlights

The therapeutic mechanism of BYQH decoction on LSCC is analyzed by network pharmacology. Sitosterol and quercetin were key active ingredients BYQH decoction. *TP53*, *EGFR*, *NOS3* and *IL1B* may be therapeutic targets of LSCC.

## RESULTS

### Active chemical compounds of BYQH decoction

**Figure 1:**
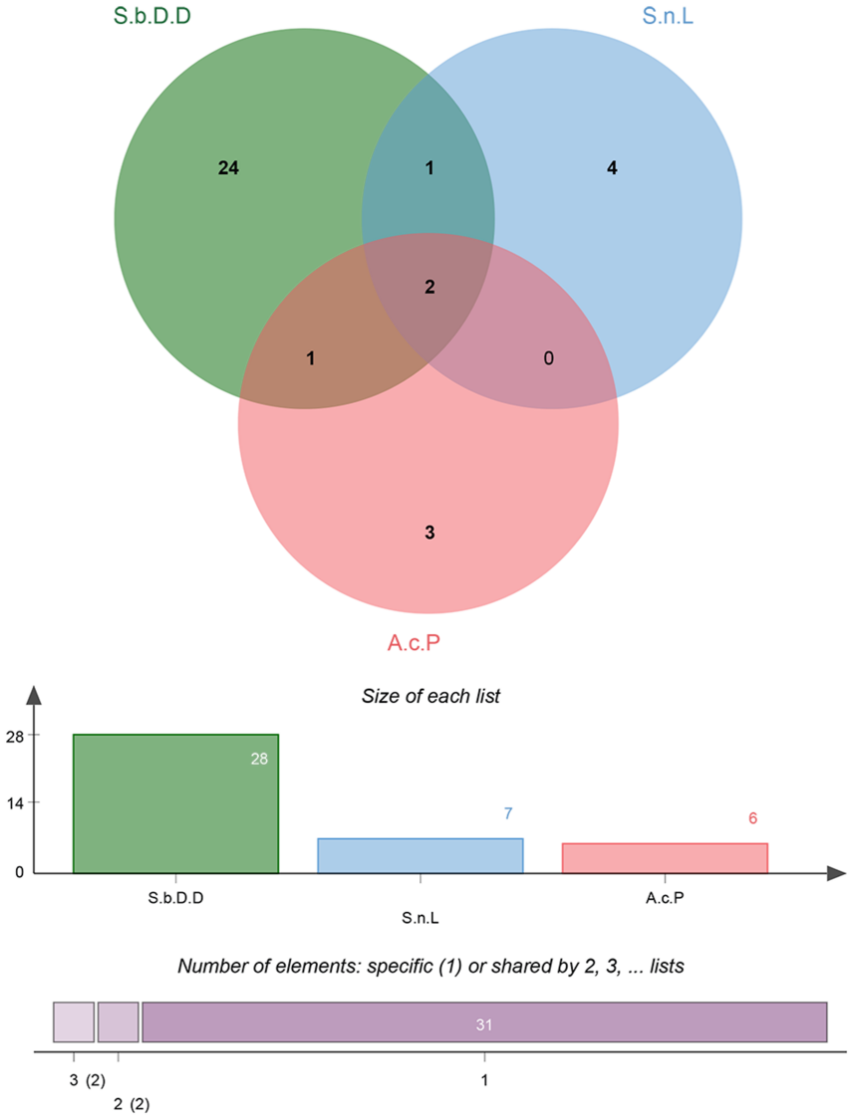
**Venn diagrams for active ingredients of Chinese herbal medicine composition in Baiying Qinghou decoction.** Abbreviations: S.b.D.D: Scutellaria barbata D. Don; S.n.L: Solanum nigrum L; A.c.P: Actinidia chinensis Planch.

The active chemical compounds of BYQH were retrieved from TCMSP database by using five keywords of “Solanum lyratum thumb” or “Scutellaria barbata” or “Duchesnea indica” or “Solanum nigrum” or “Actinidia chinensis Planch”. Totally, 174 active ingredients were obtained: 15 in Solanum lyratum thumb, 39 in Solanum nigrum, 94 in Scutellaria barbata and 26 in Actinidia chinensis Planch, respectively. Notably, the active components of Duchesnea indica were not included in TCMSP database. Subsequently, 41 key active compounds (7 in Solanum nigrum, 28 in Scutellaria barbata and 6 in Actinidia chinensis Planch) were further screened according to the ADME parameters of OB ≥ 30%, DL ≥ 0.18 and Caco-2 ≥ −0.4 (Table 1; Figure 1). Interestingly, sitosterol and quercetin were identified in three Chinese herbs (Solanum nigrum, Scutellaria barbata and Actinidia chinensis Planch; Table 1). In addition, beta-sitosterol was identified in Scutellaria barbata and Actinidia chinensis Planch, and cholesterol was identified in Solanum nigrum and Scutellaria barbata (Table 1).

**Table 1:** The list of active components of Baiying Qinghou decoction.

**Compounds**	**Mol ID**	**Molecule Name**	**MW**	**AlogP**	**Hdon**	**Hacc**	**OB (%)**	**Caco-2**	**BBB**	**DL**	**HL**
S.b.D.D	MOL001040	(2R)-5,7-dihydroxy-2-(4-hydroxyphenyl)chroman-4-one	272.27	2.3	3	5	42.36	0.38	−0.48	0.21	16.83
S.b.D.D	MOL012245	5,7,4′-trihydroxy-6-methoxyflavanone	302.3	2.28	3	6	36.63	0.43	−0.32	0.27	16.12
S.b.D.D	MOL012246	5,7,4′-trihydroxy-8-methoxyflavanone	302.3	2.28	3	6	74.24	0.37	−0.43	0.26	16.85
S.b.D.D	MOL012248	5-hydroxy-7,8-dimethoxy-2-(4-methoxyphenyl)chromone	328.34	2.82	1	6	65.82	0.85	0.07	0.33	16.41
S.b.D.D	MOL012250	7-hydroxy-5,8-dimethoxy-2-phenyl-chromone	298.31	2.84	1	5	43.72	0.96	0.22	0.25	16.77
S.b.D.D	MOL012251	Chrysin-5-methylether	268.28	2.85	1	4	37.27	0.91	0.16	0.2	17.24
S.b.D.D	MOL012252	9,19-cyclolanost-24-en-3-ol	426.8	7.55	1	1	38.69	1.45	1.16	0.78	5.41
S.b.D.D	MOL012254	campesterol	400.76	7.63	1	1	37.58	1.34	0.98	0.71	4.63
S.b.D.D	MOL000953	CLR	386.73	7.38	1	1	37.87	1.43	1.13	0.68	4.52
S.b.D.D	MOL000358	beta-sitosterol	414.79	8.08	1	1	36.91	1.32	0.99	0.75	5.36
S.b.D.D	MOL012266	rivularin	344.34	2.55	2	7	37.94	0.65	−0.13	0.37	16.25
S.b.D.D	MOL001973	Sitosterol acetate	456.83	8.46	0	2	40.39	1.39	1.11	0.85	6.34
S.b.D.D	MOL012269	Stigmasta-5,22-dien-3-ol-acetate	454.81	8.02	0	2	46.44	1.41	1.06	0.86	6.77
S.b.D.D	MOL012270	Stigmastan-3,5,22-triene	394.75	8.43	0	0	45.03	1.9	1.81	0.71	6.21
S.b.D.D	MOL000449	Stigmasterol	412.77	7.64	1	1	43.83	1.44	1	0.76	5.57
S.b.D.D	MOL000173	wogonin	284.28	2.59	2	5	30.68	0.79	0.04	0.23	17.75
S.b.D.D	MOL001735	Dinatin	300.28	2.32	3	6	30.97	0.48	−0.49	0.27	16.44
S.b.D.D	MOL001755	24-Ethylcholest-4-en-3-one	412.77	8.18	0	1	36.08	1.46	1.22	0.76	5.49
S.b.D.D	MOL002714	baicalein	270.25	2.33	3	5	33.52	0.63	−0.05	0.21	16.25
S.b.D.D	MOL002719	6-Hydroxynaringenin	288.27	2.03	4	6	33.23	0.27	−0.27	0.24	15.67
S.b.D.D	MOL002915	Salvigenin	328.34	2.82	1	6	49.07	0.86	−0.03	0.33	15.87
S.b.D.D	MOL000351	Rhamnazin	330.31	2.01	3	7	47.14	0.53	−0.32	0.34	13.54
S.b.D.D	MOL000359	sitosterol	414.79	8.08	1	1	36.91	1.32	0.87	0.75	5.37
S.b.D.D	MOL005190	eriodictyol	288.27	2.03	4	6	71.79	0.17	−0.54	0.24	15.81
S.b.D.D	MOL005869	daucostero_qt	414.79	8.08	1	1	36.91	1.32	0.87	0.75	5.08
S.b.D.D	MOL000006	luteolin	286.25	2.07	4	6	36.16	0.19	−0.84	0.25	15.94
S.b.D.D	MOL008206	Moslosooflavone	298.31	2.84	1	5	44.09	1.01	0.54	0.25	17.02
S.b.D.D	MOL000098	quercetin	302.25	1.5	5	7	46.43	0.05	−0.77	0.28	14.4
S.n.L	MOL002058	40957-99-1	388.45	2.12	2	7	57.2	0.49	−0.29	0.62	2.04
S.n.L	MOL002773	beta-carotene	536.96	12	0	0	37.18	2.25	1.52	0.58	4.36
S.n.L	MOL000359	sitosterol	414.79	8.08	1	1	36.91	1.32	0.87	0.75	5.37
S.n.L	MOL000546	diosgenin	414.69	4.63	1	3	80.88	0.82	0.27	0.81	4.14
S.n.L	MOL007356	solanocapsine	430.75	3.49	4	4	52.94	0.39	−0.22	0.67	7.86
S.n.L	MOL000953	CLR	386.73	7.38	1	1	37.87	1.43	1.13	0.68	4.52
S.n.L	MOL000098	quercetin	302.25	1.5	5	7	46.43	0.05	−0.77	0.28	14.4
A.c.P	MOL000358	beta-sitosterol	414.79	8.08	1	1	36.91	1.32	0.99	0.75	5.36
A.c.P	MOL000359	sitosterol	414.79	8.08	1	1	36.91	1.32	0.87	0.75	5.37
A.c.P	MOL000471	aloe-emodin	270.25	1.67	3	5	83.38	−0.12	−1.07	0.24	31.49
A.c.P	MOL000492	(+)-catechin	290.29	1.92	5	6	54.83	−0.03	−0.73	0.24	0.61
A.c.P	MOL000073	ent-Epicatechin	290.29	1.92	5	6	48.96	0.02	−0.64	0.24	0.63
A.c.P	MOL000098	quercetin	302.25	1.5	5	7	46.43	0.05	−0.77	0.28	14.4

Abbreviations: S.b.D.D: Scutellaria barbata D. Don; S.n.L: Solanum nigrum L; A.c.P: Actinidia chinensis Planch; Mol ID: molecule ID; MW: molecular weight; AlogP: lipid-water partition coefficient; Hdon/Hacc: the number of hydrogen-bonding donor or acceptor; OB: oral bioavailability; BBB: blood brain barrier; Caco-2: Caco-2 permeability; DL: drug-likeness; HL: half-life.

### Functional enrichment analysis of targets of the active Ingredients of BYQH decoction

**Figure 2:**
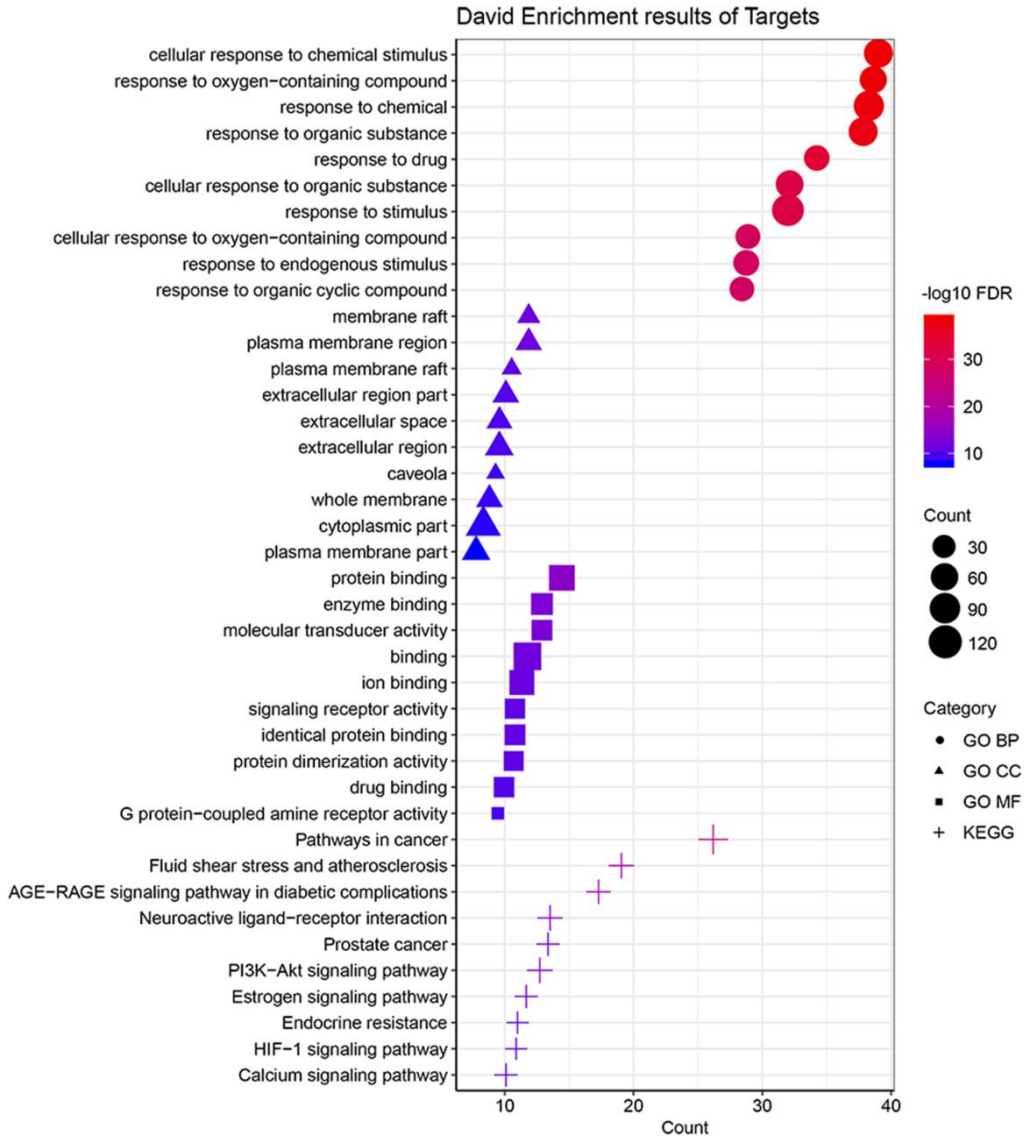
**Functional analyses of target genes of Baiying Qinghou decoction.** The top 10 significantly enriched GO-BP/MF/CC terms and KEGG pathways were displayed. Abbreviations: GO: Gene Ontology; KEGG: Kyoto Encyclopedia of Genes and Genomes; BP: biological process; CC: cellular component; MF: molecular function.

Totally, 137 gene targets of the active ingredients of BYQH decoction were predicted according to the methods mentioned above (Supplementary Table 1). Afterwards, the functional enrichment analyses of these genes were conducted to investigate their potential biological roles in LSCC treatment. The results showed that they were enriched in 1444 GO-BP terms, 114 GO-CC terms and 203 GO-MF terms. Figure 2 showed top 10 significantly enriched GO terms in MF, CC, and BP. At the cellular level, the gene targets were dramatically enriched in cellular response to chemical stimulus (involved with *TP53*, *EGFR*, *NOS3* and *IL1B* et al.), response to drug and response to chemical (involved with *TP53*, *EGFR*, *NOS3* and *IL1B* et al.). The membrane raft, plasma membrane region and plasma membrane raft were the three GO-CC terms enriched by gene targets. For GO-MF category, these genes were significantly involved in protein binding, enzyme biding and molecular transducer activity. In addition, the KEGG enrichment analysis revealed that the target genes were enriched in 172 pathways. Moreover, we found they were primarily correlated with several cancer-related pathways, such as PI3K-Akt signaling pathway (involved with BCL2, EGFR, NOS3 and TP53 et al.), estrogen signaling pathway (involved with BCL2, EGFR, MMP2, NOS3 et al.) and HIF-1 signaling pathway (involved with EGF, EGFR, IL6 and NOS3 et al.). These findings suggested that BYQH decoction may have essential effect on the treatment of LSCC.

### Target PPI network analysis

**Figure 3:**
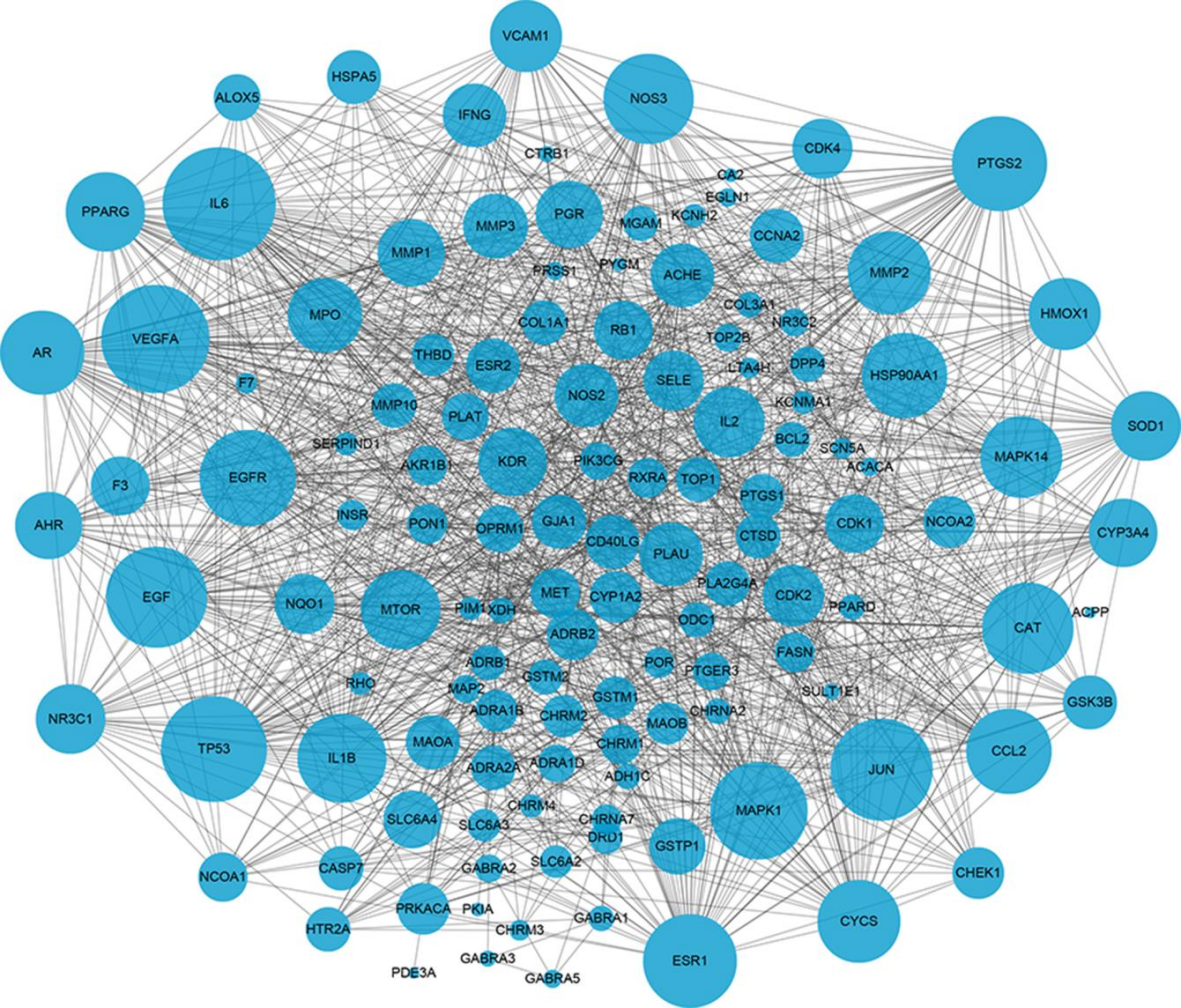
**The protein-protein interaction (PPI) network of key target genes of Baiying Qinghou decoction.** Dots represent target proteins/genes and the lines represents interactions among proteins/targets. The larger dot size shows the higher degree.

The potential interactions among target proteins of BYQH decoction was constructed and visualized by STRING database. As shown in Figure 3, there were 128 protein nodes and 1282 edges in PPI network. The protein nodes with higher degree may have closer biological connection with other nodes. Herein, the top 15 nodes were regarded as hub genes and listed in Table 2, including *IL6* (interleukin-6 degree = 67), *VEGFA* (vascular endothelial growth factor A; degree = 63), *TP53* (degree = 61), *JUN* (degree = 58), *EGF* (degree = 57), *MAPK1* (degree = 54), *EGFR* (degree = 53), *PTGS2* (degree = 52), *ESR1* (degree = 51), *CAT* (degree = 49), *NOS3* (degree = 48), *IL1B* (degree = 47), *HSP90AA1* (degree = 44), *CCL2* (degree = 44), and *AR* (degree = 43).

**Table 2:** The top 15 hub proteins in protein-protein interaction network.

**Name**	**Degree**	**Betweenness centrality**	**Closeness centrality**
IL6	67	0.080	0.658
VEGFA	63	0.050	0.638
TP53	61	0.031	0.608
JUN	58	0.043	0.626
EGF	57	0.058	0.623
MAPK1	54	0.022	0.593
EGFR	53	0.035	0.608
PTGS2	52	0.030	0.599
ESR1	51	0.031	0.585
CAT	49	0.058	0.591
NOS3	48	0.063	0.599
IL1B	47	0.021	0.593
HSP90AA1	44	0.023	0.561
CCL2	44	0.013	0.559
AR	43	0.029	0.555

### Cross-validation of target proteins

**Figure 4:**
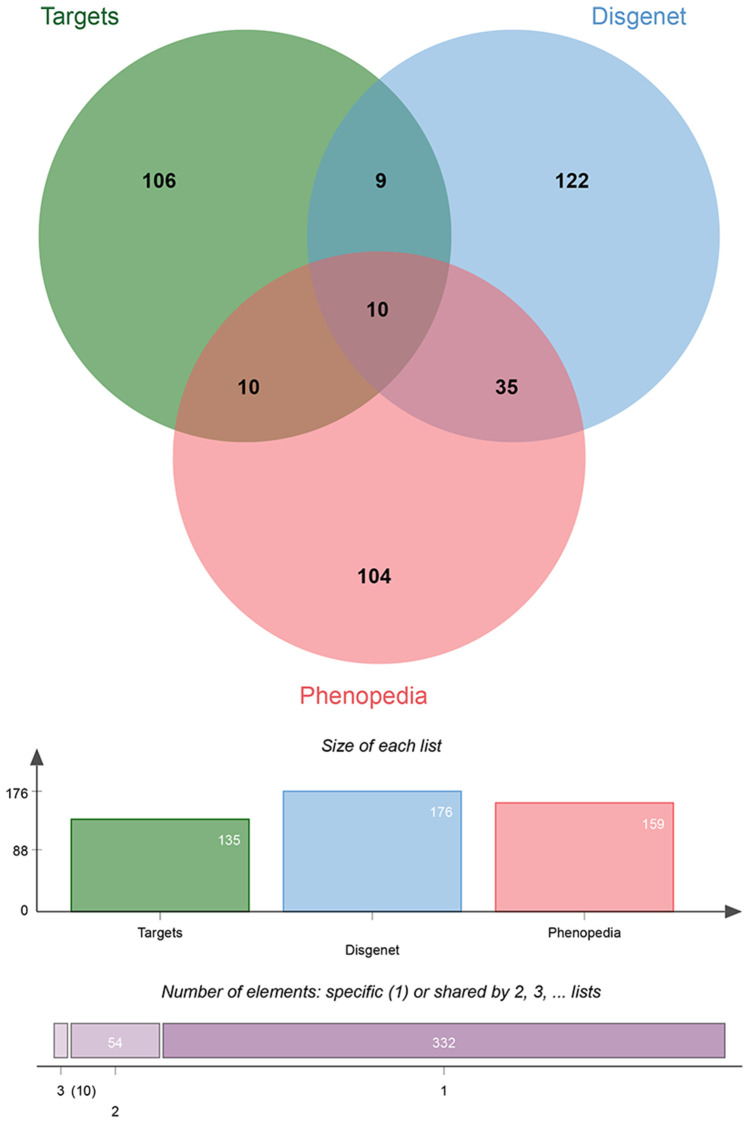
**Venn diagrams for cross-validation gene targets by Phenopedia and DisGeNET databases.** The intersecting genes were extracted between gene targets of Baiying Qinghou decoction and genes related to laryngeal cancer in Phenopedia and DisGeNET databases.

To further narrow the range of potential target genes of BYQH decoction, the candidate target genes were cross-validated. The genes related to LSCC were respectively acquired from Phenopedia and DisGeNET database, which provided information on gene-disease interactions. Then, the intersecting target genes were obtained between predicted genes and gene targets of BYQH decoction. Finally, a total of 10 intersection-associated genes were extracted, including *PTGS2*, *NOS3*, *BCL2*, *ADH1C*, *TP53*, *MPO*, *EGFR*, *GSTP1*, *IL1B* and *GSTM1* (Figure 4). Among them, five genes (*TP53*, *EGFR*, *PTGS2*, *NOS3* and *IL1B*) served as the hub genes in PPI network. Therefore, these genes were considered to be key targets of BYQH decoction against LSCC.

### Survival analysis

**Figure 5:**
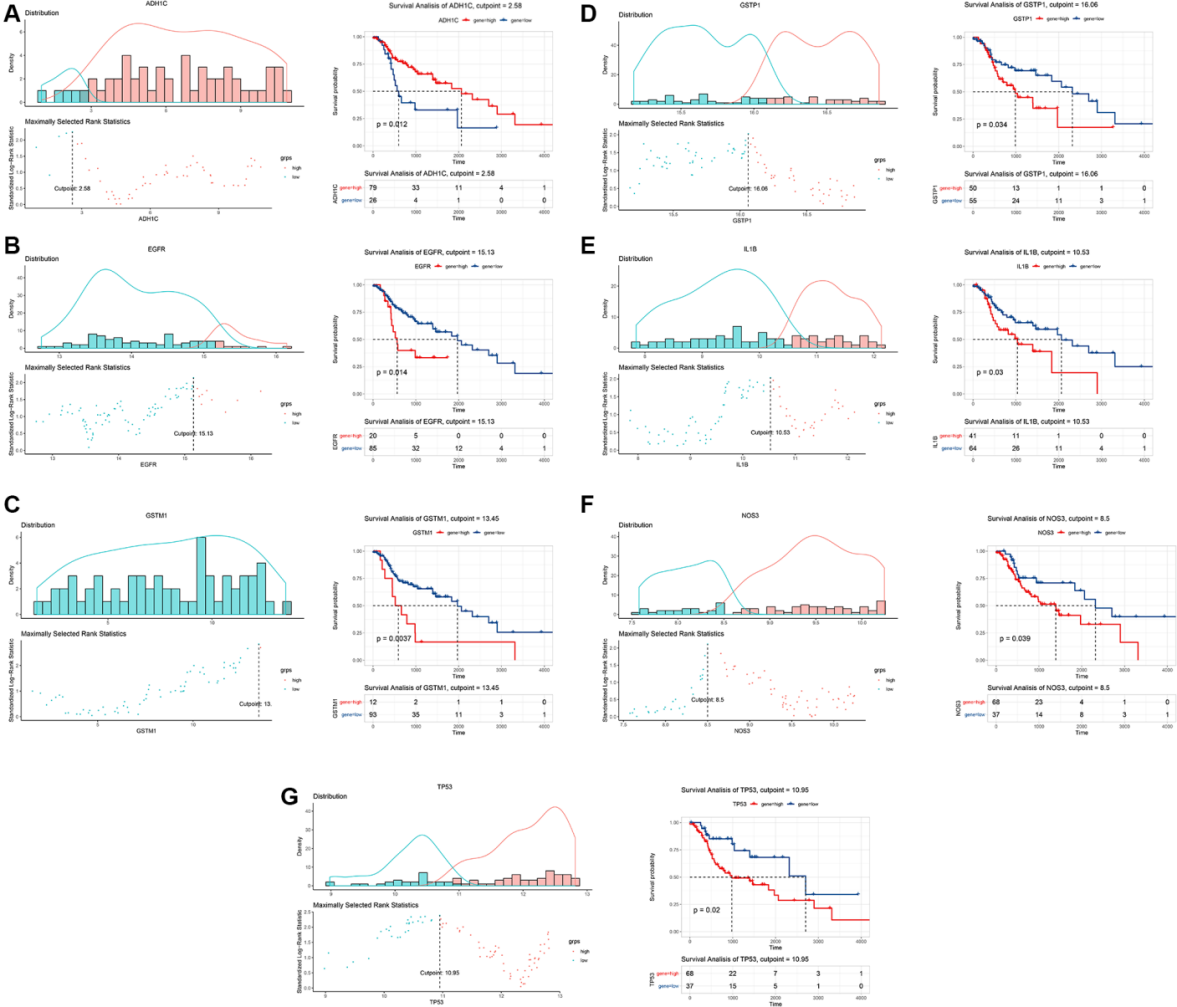
**Survival analysis of key target genes of Baiying Qinghou decoction using a TCGA dataset.** The optimal cutoff value of each gene was calculated to stratify all patients into high- and low-expression groups (the left of the **A**–**G**). Seven genes (*ADH1C*, *EGFR*, *GSTM1*, *GSTP1*, *IL1B*, *NOS3* and *TP53*) were significantly associated with the prognosis of HNSCC patients (the right of the **A**–**G**). The high expression level of *ADH1C* (**A**) had a better prognosis of HNSCC patients (*P* = 0.012). However, the high expression levels of *EGFR* (**B**), *GSTM1* (**C**), *GSTP1* (**D**), *IL1B* (**E**), *NOS3* (**F**) and *TP53* (**G**) showed the poor prognosis of HNSCC patients. Abbreviations: HNSCC: head and neck squamous cell carcinoma; TCGA: The Cancer Genome Atlas.

To explore the underlying impacts of 10 key gene targets on the clinical prognosis of LSCC, we conducted the survival analyses using TCGA data of 105 HNSCC patients. The expression patterns of these genes were firstly determined among tissue samples of 105 HNSCC patients. Then, we calculated the optimal cutoff value of each gene to stratify all patients into high- and low-expression groups. Our results showed that the optimal cutoff value was 2.58 for *ADH1C*, 7.03 for *BCL2*, 15.13 for *EGFR*, 13.45 for *GSTM1*, 16.06 for *GSTP1*, 10.53 for *IL1B*, 5.29 for *MPO*, 8.5 for *NOS3*, 9.36 for *PTGS2*, and 10.95 for *TP53* (Figure 5 and Supplementary Figure 1). Survival analysis revealed that seven genes (*ADH1C*, *EGFR*, *GSTM1*, *GSTP1*, *IL1B*, *NOS3* and *TP53*) were significantly associated with the prognosis of HNSCC patients (*P* < 0.05; Figure 5). Moreover, HNSCC patients with a high expression level of *ADH1C* had a better prognosis (*P* = 0.012; Figure 5). However, the high expression levels of the remaining six genes were closely related to the unfavorable prognosis of HNSCC patients (*EGFR*: *P* = 0.014; *GSTM1*: *P* = 0.004; *GSTP1*: *P* = 0.034; *IL1B*: *P* = 0.03; *NOS3*: *P* = 0.039 and *TP53*: *P* = 0.02; Figure 5). Notably, *TP53*, *EGFR*, *NOS3* and *IL1B* also acted as hub genes in PPI network.

### Prediction of the binding mode between key bioactive compounds and proteins

**Figure 6:**
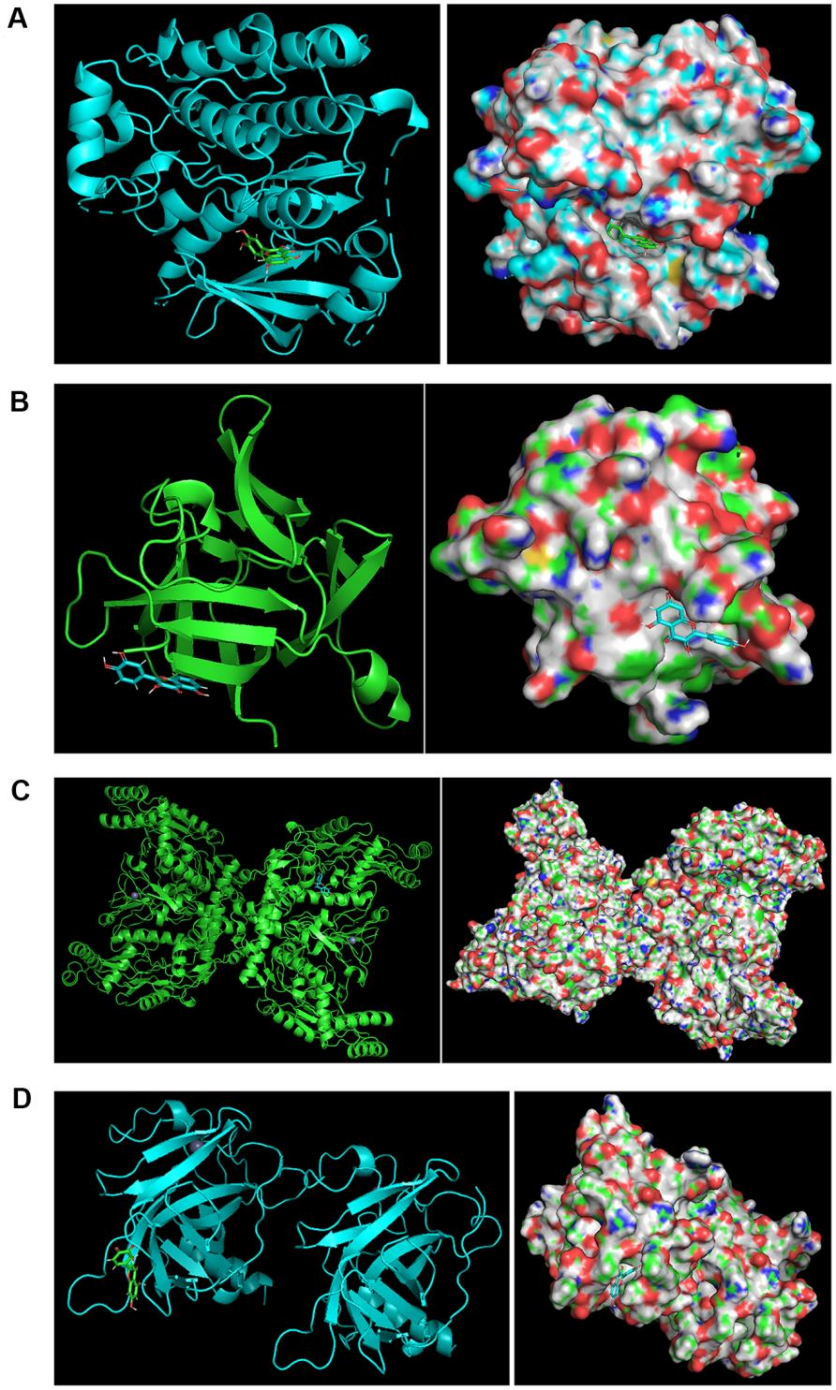
**The binding modes.** (**A**) The binding modes of quercetin and *EGFR*. The optimal binding modes of quercetin and *EGFR* (ΔG = −6.87 kcal/mol). Left: two-dimensional image; Right: three-dimensional image. (**B**) The binding modes of quercetin and *IL1B*. The optimal binding modes of quercetin and *IL1B* (ΔG = −4.84 kcal/mol). Left: two-dimensional image; Right: three-dimensional image. (**C**) The binding modes of quercetin and *NOS3*. The optimal binding modes of quercetin and *NOS3* (ΔG = −7.12 kcal/mol). Left: two-dimensional image; Right: three-dimensional image. (**D**) The binding modes of quercetin and *TP53*. The optimal binding modes of quercetin and *TP53* (ΔG = −6.15 kcal/mol). Left: two-dimensional image; Right: three-dimensional image.

The binding characteristics of key active ingredients of BYQH decoction and four key protein targets (*TP53*, *EGFR*, *NOS3* and *IL1B*) were investigated. Herein, we focused on analyzing the binding modes between two overlapped active compounds (sitosterol and quercetin) among three Chinese herbs and four target proteins. Our analysis indicated that the corresponding protein targets of sitosterol were PGR, NCOA2, NR3C2, which were not targets studied. For quercetin, 75 target proteins were obtained, including four key protein targets. Subsequently, the chemical structure formula of quercetin was acquired from TSMCP database, and four target proteins were also searched from PDB database, including TP53 (PDB ID: 3ZME), EGFR (PDB ID: 1XKK), NOS3 (PDB ID: 6PP1) and IL1B (PDB ID: 5R86). Finally, the molecular docking was carried out using LeDock tool. Notably, a closer binding between proteins and small biological molecules indicated more energy released and a lower ΔG value. Figure 6 depicted the optimal binding modes of quercetin and its four target proteins.

## DISCUSSION

TCM is an accepted medical practice and has been used for the treatment of complicated diseases such as cancers over past few decades in China [27, 28]. BYQH decoction, a TCM prescription, has been clinically proven to be effective as adjuvant treatment against LSCC. In this study, we performed a network pharmacological analysis to explore pharmacodynamic effects and therapeutic mechanism of BYQH decoction for LSCC. We identified 41 key active ingredients of BYQH decoction. Of these, sitosterol and quercetin were intersected among three Chinese herbs of BYQH decoction. Moreover, these crucial components were corresponded to 137 target proteins. Top 15 genes (*IL6*, *VEGFA*, *TP53*, *JUN*, *EGF*, *MAPK1*, *EGFR*, *PTGS2*, *ESR1*, *CAT*, *NOS3*, *IL1B*, *HSP90AA1*, *CCL2* and *AR*) were considered as hub nodes in PPI network. Among them, five proteins (*TP53*, *EGFR*, *PTGS2*, *NOS3* and *IL1B*) were cross-validated. Additionally, survival analysis showed the expressions of *ADH1C*, *EGFR*, *GSTM1*, *GSTP1*, *IL1B*, *NOS3* and *TP53* were markedly correlated with the prognosis of HNSCC patients. Finally, the binding modes of quercetin and four target proteins (*TP53*, *EGFR*, *NOS3* and *IL1B*) were constructed.

Quercetin (3,3′,4′,5,7-pentahydroxyflavone), a common flavonol, is widely distributed in plant species, such as vegetables and grains. Our results showed that quercetin was a key active chemical compound of BYQH decoction. Extensive evidence suggests that quercetin exerts diverse biological functions, such as antioxidant, anti-inflammatory, anti-bacterial, antiviral and anticarcinogenic properties [29–31]. Numerous studies have evaluated the anticarcinogenic effects of quercetin and indicated that quercetin was involved in regulating several cancer-related pathways, such PI3K/Akt/ signaling pathways and MAPK/ERK1/2 pathways [32, 33]. Sharma et al. report that quercetin induced human laryngeal HeP2 cells death and synergistically enhanced the antiproliferative ability of cisplatin in these cells [34]. Numerous researchers demonstrate that quercetin (50 μM) can significantly increase photodynamic therapy-induced cytotoxicity via reducing the cell viability of human larynx carcinoma cells (HEp-2) [35]. In addition, the apoptotic events are increased stronger in HEp-2 cells with the combination of quercetin than the single drug treatment [36]. These evidences reveals that quercetin may be a promising therapeutic molecule for LSCC treatment.

*TP53*, *EGFR*, *NOS3* and *IL1B* were key hub genes in PPI network of target proteins of BYQH decoction and cross-validated in two databases (Phenopedia and DisGeNET). More notably, these four proteins were also target proteins of quercetin. p53 protein encoded by *TP53* gene functions as a tumor-suppressive factor by regulating multiple cellular processes such as cell-cycle progression and apoptosis [37]. Overwhelming evidence suggests several mutations in *TP53* can influence the progression of HNSCC and clinical treatment response [38, 39]. Clemente-Soto AF et al. point out that quercetin can induce G2 phase cell cycle arrest and apoptosis in human cervical cancer cells, accompanied by upregulating p53 level [40]. Our molecular docking analysis suggested that quercetin can strongly bind with TP53. *EGFR* is frequently reported to be associated with HNSCC [41]. Chan et al. indicate that quercetin may suppress cell migration and invasion of HNSCC cells overexpressing *EGFR* by down-regulating the expression of *MMP-2* and *MMP-9* [42]. Similarly, Chung et al. indicate that there is a high frequency of *EGFR* copy number in HNSCC, which acts as a poor predictor for the prognosis of HNSCC patients [43]. *IL1B* is an inflammatory cytokine gene and acts as a therapeutic target for the treatment of head and neck cancer [44, 45]. NOS3 is located on chromosome 7 (7q36) and regulates at transcriptional and post-transcriptional levels [46]. Previous studies identified numerous polymorphic sites of *NOS3* such as single nucleotide polymorphism and insertion or deletion [47, 48]. Moreover, the *NOS3* polymorphisms play crucial roles in the molecular mechanism of cancers and clinical survivals of patients undergoing cancers, including laryngeal cancer [49–51]. Guo et al. recently report that quercetin has a binding interaction with *NOS3*, which was consistent with our finding [52]. Besides, we also found the elevated level of NOS3 represented an unfavorable prognosis.

Besides, in this study, GO enrichment analysis showed that the four genes of interest (*TP53*, *EGFR*, *NOS3* and *IL1B)* were significantly enriched in cellular response to chemical stimulus, response to chemical, response to drug related biological processes. TP53 is a short-lived protein that plays a central role in mediating cellular response to stressful and genotoxic stimuli, such as anticancer drugs exposure [53]. *EGFR* has been proposed to be the prognostic marker for HNSCC that closely related with radiation sensitivity, tumor size and recurrence [54]. Additionally, our survival data showed that the high expression of *TP53*, *EGFR*, *NOS3* and *IL1B* in HNSCC patients exhibited a poor prognosis, which was consistent with the previous reports mentioned above. Thus, we speculated that the quercetin might reduce drug resistance of HNSCC by targeting *TP53*, *EGFR*, *NOS3* and *IL1B.*

Furthermore, PI3K-AKT signaling is considered to be a classical pathway related with various cancers [55, 56]. The descending activation of PI3K-AKT signaling is closely associated with radiosensitivity enhancement of LSCC patients [57]. The reduced protein expressions of PI3K and AKT are accompanied by suppressed LSCC tumor growth [58]. In this study, PI3K-AKT signaling was identified to a significant pathway involved with *TP53*, *EGFR*, *NOS3*. Our findings supported the significant role of PI3K-AKT signaling pathway in LSCC and proposed the target role of the key proteins identified in this study.

Although our study identified several key components and target proteins of BYQH decoction, relevant experimental assays are needed to validate our findings. Although were considered as the prognostic indicators for patients with LSCC, The expression patterns of the four candidate prognostic indicators in LSCC have not been investigated in clinical samples. Moreover, the relationships of these prognostic genes and some clinical parameters are also not analyzed. Additionally, many chemical compounds are possibly not identified because the limited data is available in public databases, which may lead to some false positive results.

## CONCLUSIONS

Network pharmacology analysis was carried out to elucidate the therapeutic role of BYQH decoction on LSCC. Quercetin was predicted to be the active compounds of BYQH decoction by targeting *TP53*, *EGFR*, *NOS3* and *IL1B* involved in drug resistance and PI3K-AKT signaling pathway. Moreover, the four genes of *TP53*, *EGFR*, *NOS3* and *IL1B* exerted promising prognostic potential for LSCC. Our results offered an important clue for uncovering the underlying mechanism of BYQH decoction in the treatment of LSCC. However, more experimental evidences about the effects of quercetin on LSCC *in vivo* and *in vitro* by targeting *TP53*, *EGFR*, *NOS3* and *IL1B* are warranted in the near future.

## Supplementary Materials

Supplementary Figure 1

Supplementary Table 1
