# Supplementary material for: Network pharmacology reveals the potential mechanism of Baiying Qinghou decoction in treating laryngeal squamous cell carcinoma
Source: Aging (Albany NY). 2021 Dec 20;13(24):26003–21. doi: 10.18632/aging.203786 (PMC8751612; doi:10.18632/aging.203786)
Supplement: Supplementary Figure 1 [file aging-13-203786-s001.pdf]

SUPPLEMENTARY FIGURE

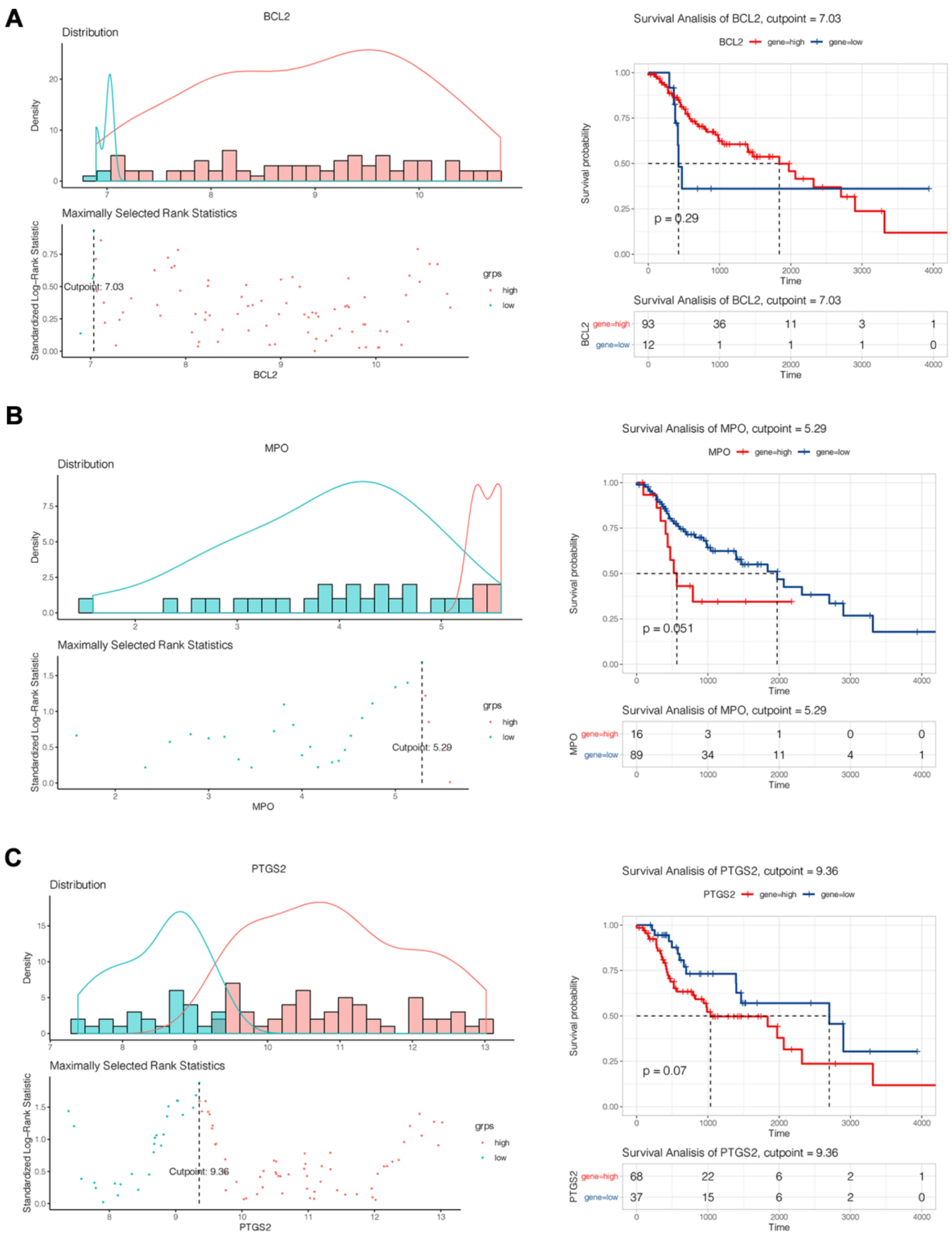

Supplementary Figure 1. Survival analysis of three target genes of Baiying Qinghou decoction using a TCGA dataset. The optimal cutoff value of each gene was calculated to stratify all patients into high- and low-expression groups (left). BCL2 (**A**), MPO (**B**), and PTGS2 (**C**) all were not related to the prognosis of HNSCC patients (*P* > 0.05; right). Abbreviations: HNSCC: head and neck squamous cell carcinoma; TCGA: The Cancer Genome Atlas.
