# Supplementary material for: Network pharmacology reveals the potential mechanism of Baiying Qinghou decoction in treating laryngeal squamous cell carcinoma
Source: Aging (Albany NY). 2021 Dec 20;13(24):26003–21. doi: 10.18632/aging.203786 (PMC8751612; doi:10.18632/aging.203786)
Supplement: Supplementary Table 1 [file aging-13-203786-s002.pdf]

SUPPLEMENTARY TABLE

Supplementary Table 1. The list of target proteins of key active components in Baiying Qinghou decoction.

Target proteins	Gene symbol	Target proteins	Gene symbol
Prostaglandin G/H synthase 1	PTGS1	Chymotrypsinogen B	CTRB1
Estrogen receptor	ESR1	Beta-1 adrenergic receptor	ADRB1
Prostaglandin G/H synthase 2	PTGS2	Tumor necrosis factor	CD40LG
Heat shock protein HSP 90	HSP90AA1	Interleukin-6	IL6
mRNA of PKA Catalytic Subunit C-alpha	PRKACA	Cellular tumor antigen p53	TP53
Beta-lactamase	LACTBL1	Interstitial collagenase	MMP1
Progesterone receptor	PGR	C-C motif chemokine 2	CCL2
Mineralocorticoid receptor	NR3C2	Prostaglandin E2 receptor EP3 subtype	PTGER3
Glucocorticoid receptor	NR3C1	Acetylcholinesterase	ACHE
Carbonic anhydrase II	CA2	Rhodopsin	RHO
Calmodulin	PCP4	Ferrichrome-iron receptor	FHUA
Phosphatidylinositol-4,5-bisphosphate 3-kinase catalytic subunit, gamma isoform	PIK3CG	Vascular endothelial growth factor A	VEGFA
Nitric oxide synthase, inducible	NOS2	Cell division control protein 2 homolog	CDK1
Thrombin	SERPIND1	Myeloperoxidase	MPO
Potassium voltage-gated channel subfamily H member 2	KCNH2	Aryl hydrocarbon receptor	AHR
Androgen receptor	AR	Cytochrome c	CYCS
Sodium channel protein type 5 subunit alpha	SCN5A	Egl nine homolog 1	EGLN1
Coagulation factor Xa	F7	Proto-oncogene serine/threonine-protein kinase Pim-1	PIM1
Nitric-oxide synthase, endothelial	NOS3	Cyclin-A2	CCNA2
Alpha-1B adrenergic receptor	ADRA1B	Peroxisome proliferator activated receptor delta	PPARD
Beta-2 adrenergic receptor	ADRB2	Heme oxygenase 1	HMOX1
DNA topoisomerase II	TOP2B	NAD(P)H dehydrogenase [quinone] 1	NQO1
Estrogen receptor beta	ESR2	Epidermal growth factor receptor	EGFR
Dipeptidyl peptidase IV	DPP4	72 kDa type IV collagenase	MMP2
Glycogen phosphorylase, muscle form	PYGM	Mitogen-activated protein kinase 1	MAPK1
Glycogen synthase kinase-3 beta	GSK3B	Retinoblastoma-associated protein	RB1
Cell division protein kinase 2	CDK2	Cell division protein kinase 4	CDK4
Serine/threonine-protein kinase Chk1	CHEK1	Xanthine dehydrogenase/oxidase	XDH
Ig gamma-1 chain C region	IGHG1	DNA topoisomerase 1	TOP1
Trypsin-1	PRSS1	Amyloid beta A4 protein	APBA3
Nuclear receptor coactivator 2	NCOA2	Caspase-7	CASP7
Nuclear receptor coactivator 1	NCOA1	Interleukin-2	IL2
Calcium-activated potassium channel subunit alpha 1	KCNMA1	Interferon gamma	IFNG
Peroxisome proliferator activated receptor gamma	PPARG	Glutathione S-transferase P	GSTP1
Retinoic acid receptor RXR-alpha	RXRA	Insulin receptor	INSR
CGMP-inhibited 3′,5′-cyclic phosphodiesterase A	PDE3A	Hepatocyte growth factor receptor	MET
Alpha-1D adrenergic receptor	ADRA1D	Stromelysin-1	MMP3
Mitogen-activated protein kinase 14	MAPK14	Pro-epidermal growth factor	EGF
Alpha-1A adrenergic receptor	ADRA1D	NADPH--cytochrome P450 reductase	POR
Sodium-dependent serotonin transporter	SLC6A4	Ornithine decarboxylase	ODC1
Amine oxidase [flavin-containing] B	MAOB	Superoxide dismutase [Cu-Zn]	SOD1
Neuronal acetylcholine receptor protein, alpha-7 chain	CHRNA7	78 kDa glucose-regulated protein	HSPA5
cAMP-dependent protein kinase inhibitor alpha	PKIA	Acetyl-CoA carboxylase 1	ACACA

Cytochrome P450-cam	CYP101A1	Cytochrome P450 3A4	CYP3A4
Dopamine D1 receptor	DRD1	Cytochrome P450 1A2	CYP1A2
Muscarinic acetylcholine receptor M3	CHRM3	Tissue factor	F3
Muscarinic acetylcholine receptor M1	CHRM1	Gap junction alpha-1 protein	GJA1
Gamma-aminobutyric-acid receptor alpha-2 subunit	GABRA2	Interleukin-1 beta	IL1B
Muscarinic acetylcholine receptor M4	CHRM4	E-selectin	SELE
5-hydroxytryptamine 2A receptor	HTR2A	Vascular cell adhesion protein 1	VCAM1
Gamma-aminobutyric-acid receptor alpha-5 subunit	GABRA5	Estrogen sulfotransferase	SULT1E1
Gamma-aminobutyric-acid receptor alpha-3 subunit	GABRA3	Maltase-glucoamylase, intestinal	MGAM
Muscarinic acetylcholine receptor M2	CHRM2	Tissue-type plasminogen activator	PLAT
Neuronal acetylcholine receptor subunit alpha-2	CHRNA2	Thrombomodulin	THBD
Mu-type opioid receptor	OPRM1	Collagen alpha-1(I) chain	COL1A1
Gamma-aminobutyric acid receptor subunit alpha-1	GABRA1	Arachidonate 5-lipoxygenase	ALOX5
Apoptosis regulator Bcl-2	BCL2	Collagen alpha-1(III) chain	COL3A1
Transcription factor AP-1	JUN	DNA gyrase subunit B	GYRB
Serum paraoxonase/arylesterase 1	PON1	Prostatic acid phosphatase	ACPP
Microtubule-associated protein 2	MAP2	Cathepsin D	CTSD
Coagulation factor VII	F7	Glutathione S-transferase Mu 1	GSTM1
Vascular endothelial growth factor receptor 2	KDR	Glutathione S-transferase Mu 2	GSTM2
Alcohol dehydrogenase 1C	ADH1C	Stromelysin-2	MMP10
Alpha-2A adrenergic receptor	ADRA2A	Fatty acid synthase	FASN
Sodium-dependent noradrenaline transporter	SLC6A2	Catalase	CAT
Sodium-dependent dopamine transporter	SLC6A3	Cytosolic phospholipase A2	PLA2G4A
Aldose reductase	AKR1B1	Serine/threonine-protein kinase mTOR	MTOR
Urokinase-type plasminogen activator	PLAU	Amine oxidase [flavin-containing] A	MAOA
Leukotriene A-4 hydrolase	LTA4H		
